# Trends and Hotspots Concerning Macular Hole between 2002 and 2021: A 20-Year Bibliometric Study

**DOI:** 10.3390/jpm13010075

**Published:** 2022-12-29

**Authors:** Yiyang Shu, Yuting Shao, Yimin Wang, Yanlong Bi

**Affiliations:** 1 Department of Ophthalmology, Tongji Hospital, School of Medicine, Tongji University, Shanghai 200065, China; 2 Department of Ophthalmology, Shanghai General Hospital, Shanghai Jiao Tong University School of Medicine, Shanghai 200080, China; 3 Tongji Eye Institute, School of Medicine, Tongji University, Shanghai 200092, China

**Keywords:** bibliometric analysis, macular hole (MH), internal limiting membrane (ILM), pars plana vitrectomy (PPV), VOSviewer

## Abstract

Background: Macular hole (MH) can severely impair central vision. Although it can be treated with vitrectomy surgery, avoiding recurrence and improving visual acuity are still priorities to be addressed. This study aims to reveal the trends and hotspots about MH. Methods: The Web of Science Core Collection (WOSCC) was used to perform a bibliometric analysis investigating trends of MH research from 2002 to 2021. We evaluated the details of associated regions, institutions, authors, and journals. To construct and overlay network visualizations, VOSviewer software was used. Results: In total, 1518 publications were collected. Our analysis showed that MH research is becoming increasingly relevant, with Japan achieving the largest number of publications (291), largest number of citations (7745 in total), and highest h-index value (48). *Retina* published the most publications on this topic, totaling more than the next two journals combined. An analysis of keyword co-occurrence was evaluated, highlighting several novel keywords of interest, such as flap technique, transplantation, epiretinal proliferation (EP), foveal microstructure, and retinal sensitivity. Conclusions: Details on MH research were uncovered by comprehensively analyzing the global trends and hotspots over the past two decades, presenting valuable information for future MH research. Japan, the USA, and China hold leading positions in research on this topic. Amendable surgical methods are a potential focus for improving prognosis.

## 1. Introduction

The vitreomacular interface (VMI) includes vitreomacular adhesion, vitreomacular traction, and potentially macular hole (MH), arising from tissue defects that may harm the retinal internal limiting membrane (ILM) or even the photoreceptor (PR) layer [1]. The impact of MH varies widely, making it a retinal disease with a broad spectrum consisting of different subtypes, each with its own defined pathogenesis, morphology, and specific therapeutic option [2].

According to the pathogenesis, MH has been conveniently divided into two types, idiopathic MH and secondary MH. The latter is attributed to but not limited to pathologic conditions [3] such as trauma [4], high myopia [5], uveitis, macular telangiectasia [6], macular schisis [5], idiopathic retinal vasoproliferative tumor [7], proliferative diabetic retinopathy (PDR) [8], and various retinal pathologies. MH may lead to vision loss, central dark spots and metamorphopsia, which can occur suddenly or gradually [9,10]. Reports of its prevalence vary widely, from 0.02% to 0.33%, in publications [9].

In the past 20 years, a lot of MH research has been published, so exploring the hotspots and trends will better guide future research. While bibliometrics can extract quantitative data through statistical analysis of scholarly publications to measure their impact [11], none are available for the topic of MH to our knowledge. Therefore, this study aims to explore published research through a bibliometric approach to informatively visualize the current MH landscape. Our study objectively assesses publication numbers, country/region contributions, co-authorship, individual productivity, and participating journals in the MH field. These were achieved through co-citation analysis and keyword co-occurrence analysis [12].

## 2. Materials and Methods

### 2.1. Search Methods

**Figure 1:**
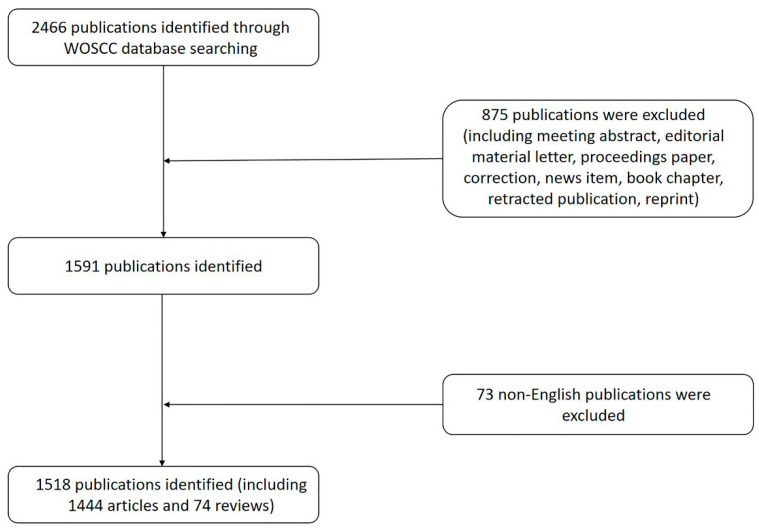
Flowchart of the search strategy of our study.

Science Citation Index Expanded in the Web of Science Core Collection (WOSCC) was used to search for publications included in this study. All the literature results included were published from 2002 to 2021 and were searched for on 12 October 2022. The keyword used was “Title = macular hole”, and 2466 results from the literature were identified. In total, 1514 articles and 77 reviews were included. Figure 1 depicts the selection process.

### 2.2. Data Collection and Bibliometric Analysis

Bibliographic information, such as author, research institution, country/region, keywords, and citations were downloaded from WOSCC databases as TXT. Network visualizations and overlay visualizations were then used for input, when data are analyzed using VOSviewer version 1.6.18 (Supported by the Centre for Science and Technology Studies of Leiden University). Microsoft Excel 2019 was used to plot graphs. Relative research interest (RRI) was calculated using the number of MH publications divided by all publications in all fields within a year. H-index was obtained from the WOSCC, for it is useful in quantifying a researcher’s output. The prediction model was f(x) = ax3 + bx2 + cx + d (x means the year and f(x) means the cumulative publications in a certain year), which was calculated using Microsoft Excel 2019.

In the network visualization, each node with a label represents an element, such as countries/regions. Occurrence frequencies such as publications are visualized using the size of nodes. Collaborative relationships between two nodes are reflected by links, with thickness and length depicting the strength of connection and relevance, respectively. Different clusters of nodes are codified using colors. In the overlay visualizations, all nodes were color-coded, highlighting the average time of appearance, written as average appearing year (AAY). Yellow means more recent, while blue means earlier appearance.

### 2.3. Research Ethics

All data in this article were searched and downloaded from a public database (WOSCC). No human subjects or animals were involved, so no ethical review was required for this study.

## 3. Results

### 3.1. Countries/Regions

**Figure 2:**
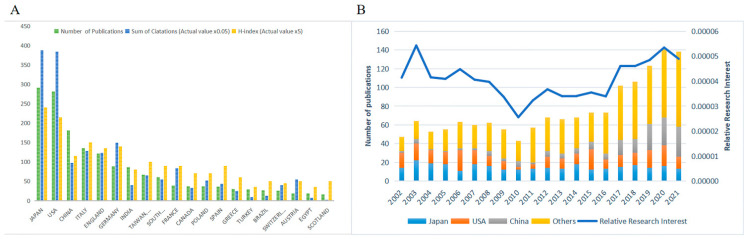
Contributions of different countries/regions. (**A**) Top 20 countries/regions in MH publications. Green bar means the number of publications, blue bar means the sum of citations in total (actual value multiplied by 0.05), and yellow bar means the h-index (actual value multiplied by 5). (**B**) The proportion and RRI of Japan, USA, China and others in the MH field from 2002 to 2021. The left axis means the number of publications, and the right axis means the RRI. RRI: relative research interest.

From 2002 to 2021, Japan contributed the most publications (291, 19.17%), followed by the USA (281, 18.51%), and then China (181, 11.92%) (Figure 2A). China in particular saw a substantial increase in MH publications. Moreover, RRI ranging from 0.003% to 0.005% indicated that the global interest has been high over the past two decades (Figure 2B).

The minimum number of documents of a country/region was set as five, and 33 countries reached that threshold out of 62. Then, we analyzed the co-authorship of the 33 countries and regions (Supplementary Figure S1); this analysis returned seven clusters. The red cluster, consisting of eight countries/regions, Egypt, India, Italy, Portugal, Saudi Arabia, Spain, Sweden, and Turkey, was the largest. We also color-coded the keywords by the AAY (Supplementary Figure S2). The yellow nodes, such as for China, India, and Italy, were the ones that published more papers recently.

### 3.2. Citations and H-Index

WOS citation reports showed that all publications related to MH have been cited 30,862 times since 2002 (16,714 citations without self-citations; 74 h-index). Based on the number of publications, the number of citations, and the h-index of each country/region, Japan (291 publications; 7745 citations in total; 48 h-index), the USA (281 publications; 7680 citations in total; 43 h-index), and China (181 publications; 1959 citations in total; 23 h-index) were the three most influential countries/regions in MH research. Of the top 10 authors, Kampik A accumulated the most citations (1712 citations in total; 1684 without self-citations; 17 h-index). Among the top 10 organizations, the University of Munich contributed the most citations (1910 citations in total; 1848 without self-citations; 18 h-index) (Table 1).

### 3.3. Publication Trends for the Future

**Figure 3:**
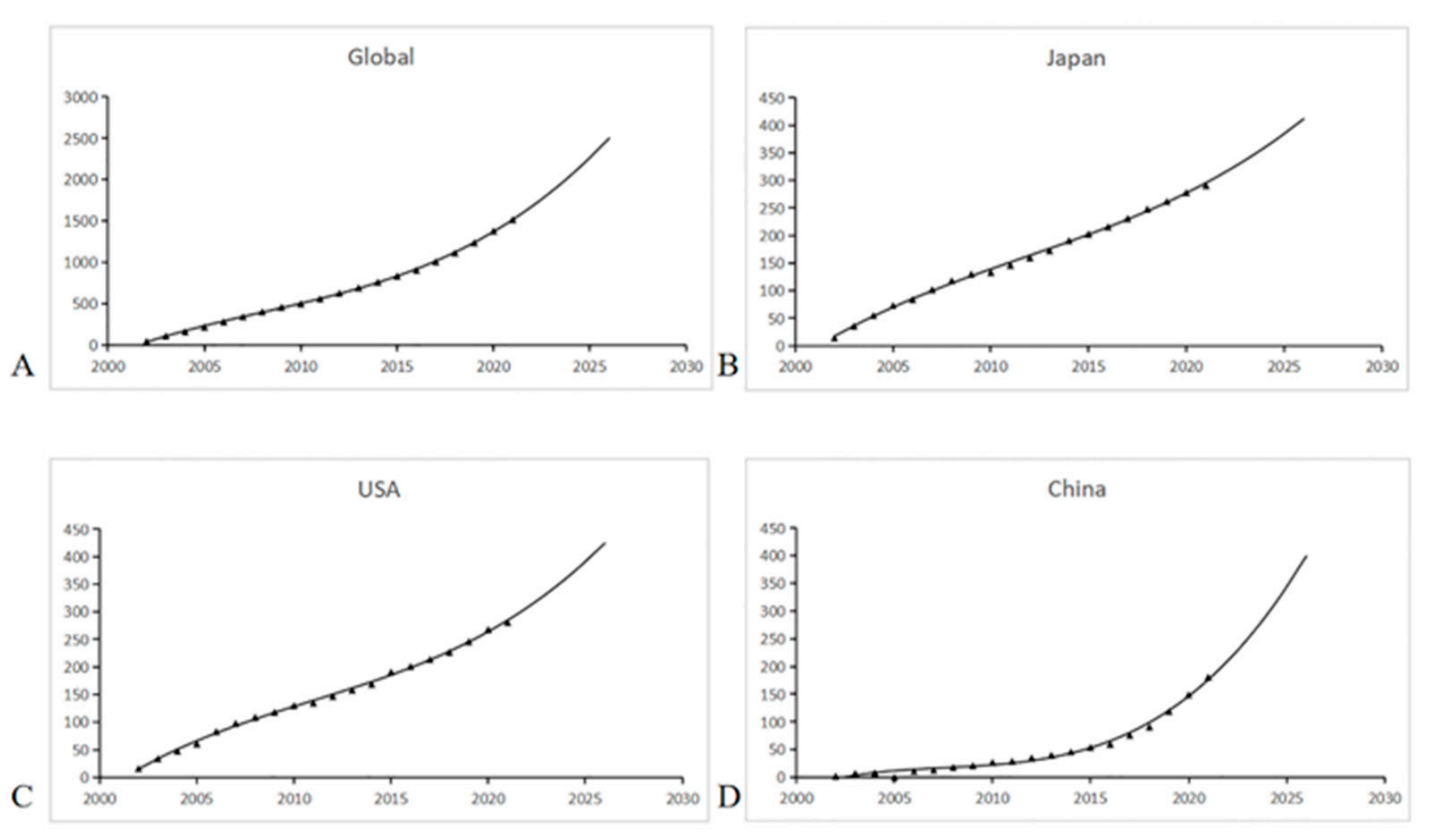
The growth and prediction curves for publications. (**A**) Global; (**B**) Japan; (**C**) the USA; (**D**) China.

The number of MH publications has continued to increase in the past two decades, and the growth curves demonstrated a significant correlation between year and cumulative publications (Figure 3). Japan and the USA held a steady and fast rate of growth (Figure 3B,C). Furthermore, the prediction model showed that China saw a much faster rate of growth since 2015 and may reach approximately 350 cumulative publications in 2025 (Figure 3D).

### 3.4. Research Organizations

The 1518 publications were written by 1405 organizations. The top 10 most productive organizations for MH research published 297 publications, accounting for 19.58% of the total publications (Table 1). The most productive organizations for MH research were the University of London (44 publications), followed by University College London (35 publications), and Moorfields Eye Hospital NHS Foundation Trust (34 publications). The co-authorship analysis network reflected the cooperation relationship among these institutions (Supplementary Figure S3).

### 3.5. Source Journals

The 1518 publications were submitted to 108 journals. Table 2 lists the top 10 active journals publishing MH research. American journals represented nearly half of all the publications included in the top 10 journals. *Retina* published the most publications (274, 18.05%), with the *American Journal of Ophthalmology* (141, 9.29%) in second place and *Graefes Archive for Clinical and Experimental Ophthalmology* (112, 7.38%) in third place.

### 3.6. Co-Authorship

**Figure 4:**
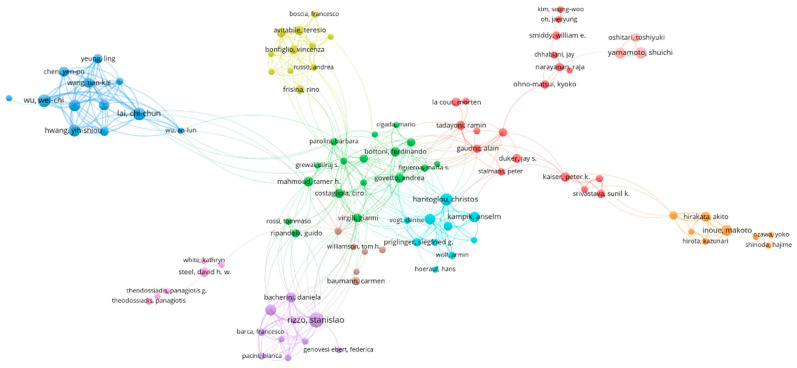
The co-authorship network. Each node with a label represents an author. The size of nodes represents the number of publications. The links between two nodes reflect the collaborative relationships between the two authors. The color of nodes means different clusters the node belongs to.

A total of 4676 authors published the 1518 retrieved publications. Table 1 lists the top 10 authors. In VOSviewer, the minimum number of publications published by an author was set as five. Of the 5284 authors, 183 authors were above that threshold. The co-authorship network, including 102 authors, was divided into 10 clusters, which are represented by different colors (Figure 4).

The top 10 papers were displayed in Table 3; the most cited paper was published in *Ophthalmology*, named The International Vitreomacular Traction Study Group Classification of Vitreomacular Adhesion, Traction, and Macular Hole. The first author was Duker, Jay S.

### 3.7. Co-Cited Authors and References

Gass J (716 co-citations) achieved the top place, followed by Michalewska Z (618 co-citations) and Kelly N (453 co-citations), among the top 10 most co-cited authors.

The minimum number of citations of a cited reference was set as 20. Of the 8988 cited references, 380 co-cited references met the threshold and were selected for analysis. References with a high similarity were found to be in seven clusters, colored in red, green, blue, yellow, orange, purple, and cyan (Supplementary Figure S4).

### 3.8. Keywords Co-Occurrence

**Figure 5:**
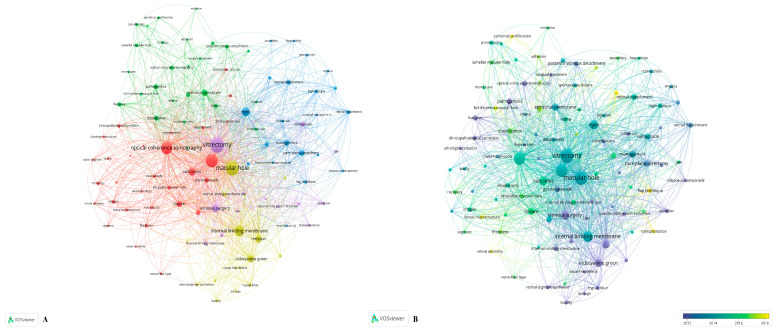
Keywords analysis by VOSviewer. (**A**) Network visualization of keywords in the field of MH research. Each color represents a single cluster, and the keywords with high similarity were clustered. (**B**) Keywords were color-coded by AAY. Yellow means more recently, and blue means earlier. AAY: average appearing year.

The minimum co-occurrence of a keyword was set as 25. Of the 2129 keywords used in MH research, 84 met the threshold and were selected for analysis. Based on this network, the keywords with a high similarity were clustered. The four main clusters are color-coded in red, yellow, green, blue, and purple (Figure 5A). Keywords were also colored according to the AAY (Figure 5B). Those colored yellow, such as flap technique, transplantation, epiretinal proliferation, foveal microstructure, and retinal sensitivity, were the ones that appeared most recently.

## 4. Discussion

### 4.1. Global Trends

Visualization of the bibliometric information was used to present a better overview of the current research status, trends, and hotspots on MH research from 2002 to 2021. In total, 1518 manuscripts were screened.

According to Supplementary Figure S1, the size of the nodes represented the publications of each country/region. The lines reflected the collaborative relationships between two nodes (the more frequent collaboration was, the thicker the connecting lines are). Although Japan had the most publications, most citations, and highest h-index, the USA engaged in the highest number of collaborations with other countries/regions. This may be attributed to the USA being the first country to perform surgical techniques for treating MH. Kelly and Wendel in the USA wrote a seminal report on the description of vitrectomy surgery for MH in 1991 [13]. In 1997, Eckardt et al. reported that ILM peeling was effective to prevent the recurrence of MH [14]. The success of closure rates has also improved with the continuous improvement of the operation [14,15,16,17,18,19,20,21,22,23,24].

The large number of surgeries performed and the development of ophthalmology in the USA have enabled many clinical studies of MH to be performed. This has contributed to the USA’s great influence in the field of MH research. Intriguingly, there were tight collaborative relationships between the top five countries/regions, while Japan and China cooperated less with others.

In total, 4676 authors from 1405 research organizations in 62 countries/regions have published publications on the topic, showing that MH research has garnered widespread interest worldwide. As shown in Supplementary Figure S3, the co-occurrence analysis network reflects the collaborative relationships between these institutions. In this network, there was extensive cooperation among organizations without geographical restrictions, while there were quite a few institutions nodes that were not linked to any other nodes. More academic cooperation and exchange between countries/regions and organizations are expected in the future.

*Retina*, the *American Journal of Ophthalmology*, and *Graefes Archive for Clinical and Experimental* were the top three journals with the highest number of publications in the field of MH research. *Retina* presented the highest number of publications, more than the second- and third-highest numbers of publications combined. This could be attributed to *Retina* being a classic journal, focused on the introduction of new surgical techniques. The main treatment for MH is surgery, which falls within the focus of *Retina*. In conclusion, the three above-mentioned journals were the three most influential journals in the field of MH research. In addition, among the top 10 most sourced journals, there were six American journals. That is to say, American journals were the leading journals on MH research by quantity.

### 4.2. Research Hotspots

The top five keywords were vitrectomy, macular hole, surgery, optical coherence tomography (OCT), and ILM. Pars plana vitrectomy (PPV) combined with ILM peeling was considered the standard procedure in the treatment of MH [25,26]. Some also suggested using gas tamponade to promote the closure of LMH [27,28]. MH formation typically evolves through four stages, which were first described by Gass [1,29]. OCT is the gold standard for MH diagnosis. In Figure 5B, yellow symbolizes more recently while blue represents earlier. Attention has been paid not only to the restoration of retinal structure but also to the restoration of visual function. Researchers are starting to focus on some novel keywords, such as flap technique, transplantation, epiretinal proliferation (EP), foveal microstructure, and retinal sensitivity.

EP is mainly composed of non-contractile GFAP-positive gliotic tissue derived from Müller cells [30]. It was first described by Witkin et al. in 2006 on an ultrahigh-resolution OCT [31]. Thus, EP could be considered a form of chronic severe gliosis in the retina. Some studies suggested that EP was a preoperative risk factor for adverse surgical outcomes [32,33,34] and could be used as a biomarker for poorer MH surgical outcomes. However, Lai et al. [35] found that preservation of EP during lamellar MH surgery was vital and could contribute to successful surgery. Therefore, EP could be one of the future hotspots in MH research. These results remind surgeons to pay close attention to EP, as it may provide a strategic approach that maximizes favorable outcomes.

However, even after the anatomical closure of MH, some patients do not experience significant improvement in visual quality. Research addressing this issue may contain keywords such as foveal microstructure and retinal sensitivity, which both place focus on the consistency of structure and function. Associations between EZ parameters and visual function have been established for several retinal diseases [36,37,38]. Foveal microstructure observations are useful for evaluating visual function.

Transplantation is another novel keyword in recent years. Although vitreous surgery combined with ILM peeling yielded a closure rate of MH as high as 90% [39,40], the risk of surgery failure might be increased, and the MH closure rate is reduced when meeting with a large MH. Aiming at a refractory larger MH, other surgical treatments should be sought, such as re-vitrectomy with extended ILM peeling [41], autologous free ILM flap transplantation [42], or transplantation of the lens capsule [43]. What do we do when an insufficient ILM or lens capsule is left? The Grewal team, thus, proposed “autologous neurosensory retinal free flap transplantation”, in 2019 [44,45]. This surgery technique has achieved a high success rate concerning outcomes that can withstand the length of time [46]. It opens up a new perspective on treatment, presenting alternatives to better cover the hole and achieve effective closure rates, such as amniotic membrane.

However, some limitations should be noted. Firstly, the bibliometrics software could not distinguish the real author contribution among complicated partnerships, which requires researchers to read the original literature themselves. Secondly, papers not recorded in WOSCC were not included, thus limiting the comprehensiveness of our study. Moreover, though our analysis was objectively completed by software, how best to interpret these results will have inherent subjective bias by the individuals involved.

## 5. Conclusions

Our study examined MH research to date and comprehensively analyzed the trends of the past two decades. In particular, Japan, the USA, and China held the leading positions in MH research. Amendable surgical methods are a potential focus for improving prognosis. With the help of information visualization analysis, clinicians are able to have a view of the current status and trends of this research field, grasp hotspots in MH research, make better clinical decisions, and predict and guide the future direction of MH research.

## Figures and Tables

**Table 1:** The top 10 productive countries/regions, organizations, and authors from 2002 to 2021.

Position	Top 10 Countries/Regions	Records	Percentage (%)	Citations	Average Citations	h-Index
1	Japan	291	19.170	7745	26.62	48
2	USA	281	18.511	7680	27.33	43
3	China	181	11.924	1959	10.82	23
4	Italy	135	8.893	2564	18.99	30
5	England	121	7.971	2466	20.38	27
6	Germany	89	5.863	2981	33.49	28
7	India	86	5.665	798	9.28	16
8	Taiwan, China	67	4.414	1308	19.52	20
9	South Korea	61	4.018	1087	17.82	18
10	France	39	2.569	1670	42.82	18
**Position 2**	**Top 10 Organizations**					
1	University of London	44	2.899	999	22.7	16
2	University College London	35	2.306	742	21.2	12
3	Moorfields Eye Hospital NHS Foundation Trust	34	2.240	739	21.74	12
4	University of Munich	31	2.042	1910	61.61	18
5	National Taiwan University	28	1.845	474	16.93	13
6	National Taiwan University Hospital	27	1.779	474	17.56	13
7	Bascom Palmer Eye Institute	25	1.647	952	38.08	14
8	National Kapodistrian University of Athens	25	1.647	418	16.72	11
9	Capital Medical University	24	1.581	218	9.08	9
10	Chang Gung Memorial Hospital	24	1.581	609	25.38	12
**Position 3**	**Top 10 Authors**					
1	Yang CM	25	1.647	451	18.04	12
2	Rizzo S	23	1.515	544	23.65	13
3	Haritoglou C	22	1.449	1466	66.64	17
4	Wu WC	22	1.449	439	19.95	12
5	Yamamoto S	20	1.318	737	36.85	13
6	Ikuno Y	19	1.252	531	27.95	14
7	Kampik A	19	1.252	1712	90.11	17
8	Lai CC	19	1.252	347	18.26	9
9	Inoue M	18	1.186	540	30	12
10	Baba T	17	1.120	795	46.76	11

**Table 2:** Top 10 source journals on MH research from 2002 to 2021.

Rank	Journal	Country /Region	Records	Percentage (%)	Journal Impact Factor in 2021
1	*Retina*	USA	274	18.050	3.975
2	*American Journal of Ophthalmology*	USA	141	9.289	5.488
3	*Graefes Archive for Clinical and Experimental Ophthalmology*	USA	112	7.378	3.535
4	*British Journal of Ophthalmology*	England	71	4.677	5.908
5	*Eye*	England	64	4.216	4.456
6	*Journal of Ophthalmology*	USA	63	4.150	1.974
7	*Ophthalmic Surgery Lasers and Imaging Retina*	USA	61	4.018	1.296
8	*European Journal of Ophthalmology*	Italy	55	3.623	1.922
9	*Ophthalmology*	USA	53	3.491	14.277
10	*BMC Ophthalmology*	England	48	3.162	2.086

**Table 3:** Top 10 most highly cited publications ranked by number of citations.

Rank	Title	First Author	Journal	Year, Volume, Issue, and Pages	Citation
1	The International Vitreomacular Traction Study Group Classification of Vitreomacular Adhesion, Traction, and Macular Hole	Duker, Jay S.	*Ophthalmology*	2013, 120 (12), 2611–2619	586
2	Inverted Internal Limiting Membrane Flap Technique for Large Macular Holes	Michalewska, Zofia	*Ophthalmology*	2010, 117 (10), 2018–2025	418
3	Enzymatic Vitreolysis with Ocriplasmin for Vitreomacular Traction and Macular Holes	Stalmans, Peter	*New England Journal of Medicine*	2012, 367 (7), 606–615	395
4	Retinal pigment epithelial changes after macular hole surgery with indocyanine green-assisted internal limiting membrane peeling	Engelbrecht, NE	*American Journal of Ophthalmology*	2002, 133 (1), 89–94	263
5	Indocyanine green-assisted peeling of the internal limiting membrane in macular hole surgery affects visual outcome: A clinicopathologic correlation	Haritoglou, C	*American Journal of Ophthalmology*	2002, 134 (6), 836–841	257
6	Macular hole size as a prognostic factor in macular hole surgery	Ullrich, S	*British Journal of Ophthalmology*	2002, 86 (4), 390–393	248
7	Prevalence and characteristics of foveal retinal detachment without macular hole in high myopia	Baba, T	*American Journal of Ophthalmology*	2003, 135 (3), 338–342	202
8	Autologous Transplantation of the Internal Limiting Membrane for Refractory Macular Holes	Morizane, Yuki	*American Journal of Ophthalmology*	2014, 157 (4), 861–869	176
9	Internal Limiting Membrane Peeling versus No Peeling for Idiopathic Full-Thickness Macular Hole: A Pragmatic Randomized Controlled Trial	Lois, Noemi	*Investigative Ophthalmology & Visual Science*	2011, 52 (3), 1586–1592	176
10	Types of macular hole closure and their clinical implications	Kang, SW	*British Journal of Ophthalmology*	2003, 87 (8), 1015–1019	175

## Data Availability

Not applicable.

## Supplementary Materials

The following supporting information can be downloaded at: https://www.mdpi.com/article/10.3390/jpm13010075/s1. Supplementary Figure S1: Network visualization of co-authorship of 33 countries and regions in the field of MH research; Supplementary Figure S2: The co-authorship map of 33 countries and regions were color-coded by AAY; Supplementary Figure S3: The co-authorship analysis network of institutions by VOSviewer; Supplementary Figure S4: The analysis of the co-cited references.
