# Supplementary material for: Trends and Hotspots Concerning Macular Hole between 2002 and 2021: A 20-Year Bibliometric Study
Source: J Pers Med. 2022 Dec 29;13(1):75. doi: 10.3390/jpm13010075 (PMC9860867; doi:10.3390/jpm13010075)
Supplement: Supplementary file 1 [file jpm-13-00075-s001.zip › jpm-2050083-supplementary.pdf]

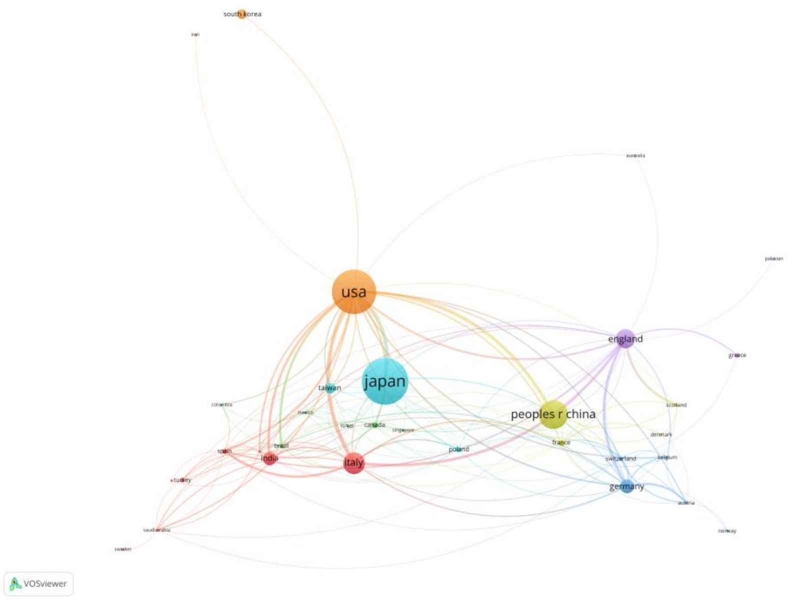

Supplementary Figure S1. Network visualization of co-authorship of 33 countries and regions in the field of MH research. The size of nodes represents publications of each country/region. Lines reflect collaborative relationships between two nodes (the more frequent collaboration, the thicker the connecting lines).

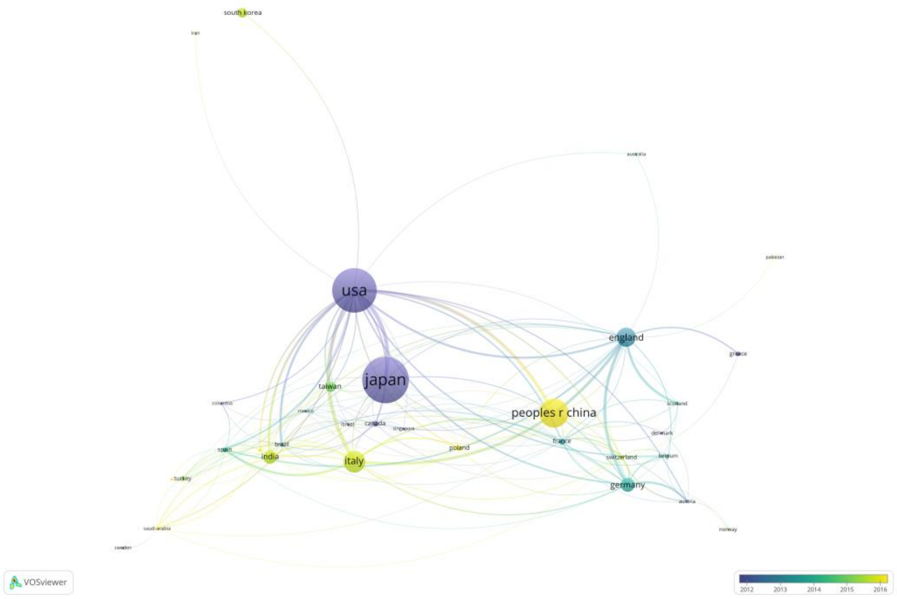

Supplementary Figure S2. The co-authorship map of 33 countries and regions were color-coded by AAY. Color intensity is related to the AAY, with yellow representing a later AAY. AAY, average appearing year.

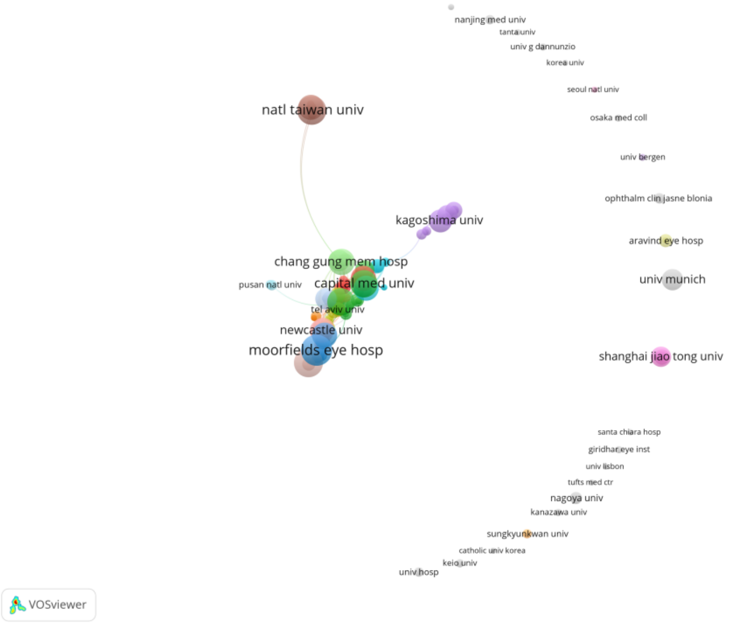

Supplementary Figure S3. The co-authorship analysis network of institutions.

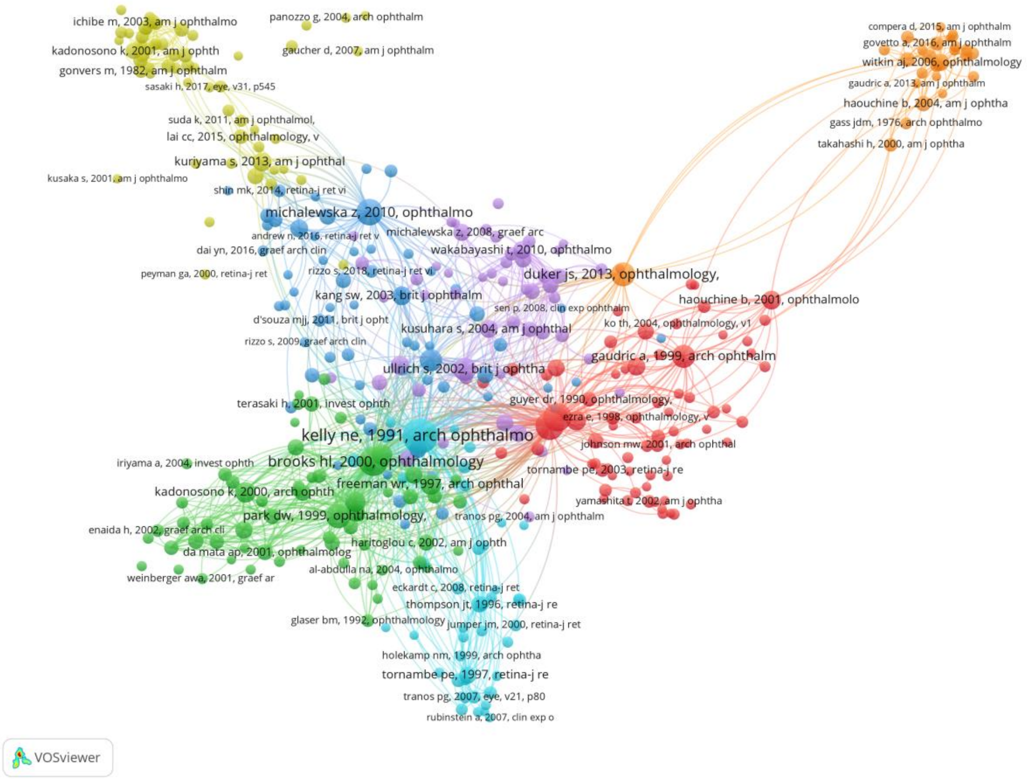

Supplementary Figure S4. The analysis of the co-cited references.
